# Changes in Functional Glucocorticoid Sensitivity of Isolated Splenocytes Induced by Chronic Psychosocial Stress – A Time Course Study

**DOI:** 10.3389/fimmu.2021.753822

**Published:** 2021-10-05

**Authors:** Elena Kempter, Mattia Amoroso, Hannah L. Duffner, Andrea M. Werner, Dominik Langgartner, Sandra Kupfer, Stefan O. Reber

**Affiliations:** Laboratory for Molecular Psychosomatics, Department of Psychosomatic Medicine and Psychotherapy, Ulm University, Ulm, Germany

**Keywords:** chronic psychosocial stress, chronic subordinate colony housing (CSC), glucocorticoid resistance, corticosterone, spleen, bite wounds

## Abstract

Chronic psychosocial stress is a risk factor for the development of numerous disorders, of which most are associated with chronic low-grade inflammation. Given the immunosuppressive effects of glucocorticoids (GC), one underlying mechanism might be the development of stress-induced GC resistance in certain immune cell subpopulations. In line with this hypothesis, male mice exposed to the chronic subordinate colony housing (CSC, 19 days) model develop GC resistance of *in vitro* lipopolysaccharide (LPS)-stimulated splenocytes, splenomegaly and an increased percentage of splenic CD11b^+^ cells. Here male C57BL/6N mice were euthanized at different days during CSC, and following 30 days of single housing after stressor termination to assess when CSC-induced splenic GC resistance starts to develop and whether this is a transient effect. Moreover, splenic CD11b, GC receptor (GR) and/or macrophage migration inhibiting factor (MIF) protein levels were quantified at respective days. While mild forms of CSC-induced GC resistance, increased splenic CD11b expression and/or splenomegaly were detectable on days 8 and 9 of CSC, more severe forms took until days 15 and 16 to develop, but normalized almost completely within 30 days following stressor termination (day 51). In contrast, splenic GR expression was decreased in CSC versus single-housed control (SHC) mice at all days assessed. While MIF expression was increased on days 15 and 16 of CSC, it was decreased in CSC versus SHC mice on day 20 despite persisting splenomegaly, increased CD11b expression and functional GC resistance. In summary, our data indicate that GC resistance and CD11b^+^ cell-mediated splenomegaly develop gradually and in parallel over time during CSC exposure and are transient in nature. Moreover, while we can exclude that CSC-induced reduction in splenic GR expression is sufficient to induce functional GC resistance, the role of MIF in CD11b^+^ cell-mediated splenomegaly and GC resistance requires further investigation.

## Introduction

Chronic psychosocial stress is a risk factor for the development of numerous somatic and affective disorders (1–3). It is further a well-known inducer of inflammatory responses and is often associated with chronic low-grade inflammation (4, 5). In turn, results from both pre-clinical and clinical studies support the hypothesis that an overshooting immune response to stress promotes the development of stress-related somatic and affective disorders (6–10). One possible mechanism underlying stress-induced chronic low-grade inflammation might be the development of glucocorticoid (GC) resistance, defined as a state of reduced sensitivity to the anti-inflammatory action of GCs in certain immune cell subpopulations (10, 11). In line with this hypothesis, isolated peripheral blood mononuclear cells (PBMC) from chronically stressed healthy teachers showed higher lipopolysaccharide (LPS)-stimulated production of interleukin (IL)-6, which was further less sensitive to the inhibiting effects of GCs (12). Moreover, animal studies indicate that isolated and LPS-stimulated splenocytes develop reduced sensitivity to the suppressive effects of GCs following several models of repeated/chronic psychosocial stress with direct physical contact between the conspecifics, including the paired fighting, social reorganization, social disruption (SDR) and chronic subordinate colony housing (CSC) paradigm (13–19), effects accompanied by pronounced splenomegaly in all mentioned stress paradigms (4, 14, 15, 19–23).

Interestingly, Avitsur and colleagues further report that physical injury in the form of bite wounds are required in order to develop SDR-induced functional GC resistance of isolated splenocytes following *in vitro* stimulation with LPS (24). Recent studies from our group have confirmed and extended those findings by showing a strong positive correlation between the development of functional splenic GC insensitivity and the severity of bite wounds received by CSC mice (14). Follow up studies from our group further revealed that mice undergoing abdominal surgery prior to chronic psychosocial stress exposure developed functional splenic GC insensitivity in a bite wound-independent manner, suggesting that not psychosocial stress *per se*, but rather the combination of psychosocial stress and any kind of physical injury drives the development of functional *in vitro* GC resistance (15, 19). In addition to the reduced sensitivity to GCs, the number of CD11b^+^ cells increases in the spleens of both SDR and CSC mice (14, 21). Interestingly, selective cell depletion studies employing both the SDR (18) and the CSC paradigm (14, 15) revealed that specifically CD11b^+^ splenocytes, and not other cell types like CD19^+^ B cells (18), are required for the development of functional GC insensitivity following LPS stimulation *in vitro*. Together with an enhanced bone marrow myelopoiesis and an increased abundance of CD11b^+^ cells in the blood of both SDR and CSC mice (14, 25–28), the above described findings suggest that stress-induced splenic functional *in vitro* GC resistance is mediated by GC-insensitive CD11b^+^ cells migrating from the bone marrow into the spleen when chronic psychosocial stress is paralleled by physical injury (15, 19).

Previous findings employing the SDR stress paradigm, which is based on the repeated social defeat of group-housed male mice in their home cage by an aggressive intruder mouse for 2 h at the beginning of the dark phase (24, 29), further showed that splenic GC resistance develops following six (29), but not one or three daily 2 h SDR sessions during the active phase of male mice (20, 21, 24). Moreover, deficits in GC sensitivity found in SDR mice seem to be transient but relatively long-lasting, persisting for at least 10 but not 30 days after the last SDR exposure (21). In contrast to the SDR paradigm, it has not been assessed so far when functional splenic *in vitro* GC resistance develops during CSC exposure and whether this is a transient process. During CSC experimental mice are housed in continuous subordination to a larger dominant male conspecific for 19 consecutive days, with the dominant male being replaced on days 8 and 15 to avoid habituation (4, 17, 22, 30).

Therefore, in the present study male C57BL/6N mice were euthanized at day 8 (right before exposure to the 2^nd^ aggressor), day 9 (24 h after exposure to the 2^nd^ aggressor), day 15 (right before exposure to the 3^rd^ aggressor), day 16 (24 h after exposure to the 3^rd^ aggressor) and day 20 of CSC to subsequently assess functional *in vitro* GC sensitivity of isolated and LPS-stimulated spleen cells, as well as spleen weight and bite wound severity. Furthermore, to assess whether CSC-induced functional *in vitro* GC resistance of isolated spleen cells is a transient or permanent consequence of stressor exposure accompanied by bite wound-induced injury, we exposed mice to 19 days of CSC and kept them single-housed (SH) for another 30 days (day 51 after being introduced to the 1^st^ aggressor) after stressor termination to assess the above detailed parameters. In order to unravel the mechanisms underlying CSC-induced functional GC resistance, we quantified splenic CD11b and GR protein levels at days 8, 9, 15, 16 and 51, as well as macrophage migration inhibiting factor (MIF) protein levels at days 8, 9, 15, 16, 20 and 51. The latter is a pro-inflammatory cytokine which has pleiotropic effects on the immune response and is able to antagonize the anti-inflammatory effects of GCs (31–33). Importantly, the present study employs male mice only, as the CSC paradigm is based on territorial aggression and chronic social subordination, features that constitute important stressors in male, but not female mice (34–36).

## Methods

### Animals

Male C57BL/6N mice weighing 19-22g were obtained from Charles River (Sulzfeld, Germany) and single housed for one week prior to the start of the experiment. Male CD-1 mice (30-35g, Charles River, Sulzfeld, Germany) were used as dominant aggressors. Mice were kept in standard polycarbonate mouse cages (16 x 22 x 14 cm) under standard laboratory conditions (12 h light/12 h dark cycle, 22°C, 60% humidity). Mice had free access to tap water and standard mouse diet. All experiments were approved by the Committee on Animal Health and Care of the local government and performed according to international guidelines on the ethical use of animals. All efforts have been made to minimize the number of animals and their suffering. Females were not used in the current study as the CSC paradigm is based on territorial aggression and the establishment of social hierarchies (4), which is not typically seen in female mice. The research described here was conducted in compliance with the ARRIVE Guidelines for Reporting Animal Research (37).

### Experimental Procedures

**Figure 1:**
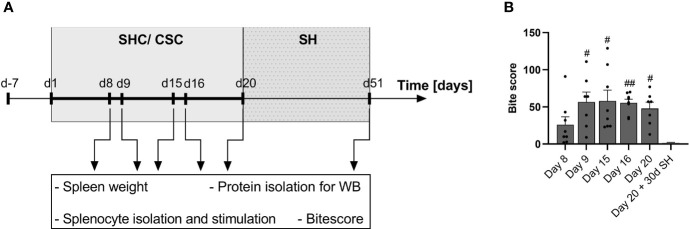
Experimental timeline and bite score. **(A)** Schematic drawing of the experimental timeline. Mice arrived on day (d) -7 and were single-housed for one week. From day 1 on, mice were either kept as single-housed controls (SHC) or exposed to the chronic subordinate colony housing (CSC) paradigm. Four CSC mice were housed together with a larger, dominant CD-1 mouse in order to induce chronic psychosocial stress. To avoid habituation, CSC mice were exposed to a novel aggressor mouse on days 8 and 15 of the CSC paradigm. Mice were sacrificed in the morning of day 8 (right before exposure to the 2^nd^ aggressor), day 9 (24 h after exposure to the 2^nd^ aggressor), day 15 (right before exposure to the 3^rd^ aggressor) and day 16 (24 h after exposure to the 3^rd^ aggressor) and day 20. One set of animals was single housed (SH) on day 20 for 30 consecutive days and then euthanized in the morning of day 51. After euthanasia, spleen weight and the severity of bite wounds were assessed. Additionally, splenocytes were isolated and stimulated, and used for further protein isolation for Western Blot (WB) analysis. **(B)** Bite score to assess the severity of bite wounds in CSC mice euthanized on days 8, 9, 15, 16 and 20 of the CSC paradigm or 30 days after stressor termination. Data are presented as mean + SEM including individual values. ^#^ *P* ≤ 0.05, ^##^ *P* ≤ 0.01 *versus* day 20 + 30d SH.

Upon arrival (day -7), all experimental mice were single housed for one week, before they were assigned to the chronic subordinate colony housing (CSC) or single-housed control (SHC) group (day 1, **Figure 1A**). To investigate the time-dependent effects of CSC on various spleen parameters (for details see below), mice were euthanized in the morning of day 8 (right before exposure to the 2^nd^ aggressor; SHC: n = 8; CSC: n = 8), day 9 (24 h after exposure to the 2^nd^ aggressor; SHC: n = 8; CSC: n = 7), day 15 (right before exposure to the 3^rd^ aggressor; SHC: n = 8; CSC: n = 8) or day 16 (24 h after exposure to the 3^rd^ aggressor; SHC: n = 8; CSC: n = 8). Another set of mice was euthanized in the morning of d20 at the end of the CSC paradigm (SHC: n = 5; CSC: n = 7). Additionally, another set of mice was single-housed for another 30 days following termination of the CSC or SHC procedure (day 20) and euthanized on day 51 (SHC: n = 8; CSC: n = 5). All experimental mice were euthanized between 06.00 and 10.00 a.m. by rapid decapitation following brief CO_2_ inhalation. Body and spleen weight, the severity of received bite wounds, splenic *in vitro* GC sensitivity under basal and LPS conditions and splenic MIF protein expression were assessed in all sets of animals. Protein expression of CD11b and GR were assessed in the spleens of animals sacrificed at days 8, 9, 15, 16 and 51. Moreover, at days 20 and 51 adrenal glands were removed and pruned from fat to assess absolute and relative adrenal weight. According to the Grubbs Test, absolute spleen weight of one SHC mouse euthanized on day 9 was excluded (SHC: n = 7 for absolute spleen weight). Additionally, the spleen of one CSC mouse euthanized on day 16 was not weighed (CSC: n = 7 for absolute and relative spleen weight). Of note, parameters related to the time course of CSC-induced changes in hypothalamus-pituitary-adrenal (HPA) axis activity (i.e. plasma adrenocorticotropic hormone (ACTH) and corticosterone concentrations, adrenal *in vitro* ACTH sensitivity, adrenal weight) assessed in the same experimental mice euthanized on days 8, 9, 15 and 16 of the current study have been reported earlier by our group (38).

### Chronic Subordinate Colony Housing (CSC) Procedure

The CSC paradigm was conducted as previously described (4, 22, 30, 39, 40). Briefly, on day 1 all experimental mice were assigned to either the CSC or the SHC group in a body weight-matched manner. Afterwards, four experimental CSC mice were housed together with a dominant CD-1 male mouse for 19 consecutive days, in order to induce a chronic stressful situation. Before the CSC procedure, the future dominant males were tested for their aggressive behavior and mice that injured their opponents by excessive aggression were excluded. Notably, the number of bite wounds received by the residents could thereby be reduced, but not totally prevented. To avoid habituation, each dominant male was replaced by a novel dominant male at days 8 and 15 of the CSC procedure. SHC mice remained undisturbed in their home cages except for change of bedding once a week. Based on our previous data indicating pronounced social hierarchy effects on physiological and behavioral parameters when group housing non-familiar same-size male conspecifics, single housing and not group housing is considered to represent the most appropriate housing condition for controls in this paradigm (41).

### Assessment of the Severity of Bite Wounds

The severity of bite wounds in CSC mice was assessed using a novel bite score previously established by our group (14). Briefly, following decapitation, the skin (with fur attached) was removed so that skin and body were still connected at the tail and hind limbs. Afterwards, pictures were taken from each mouse and digitally overlaid with a standardized grid, which contained 20 squares covering the skin (dermal wounds) and 20 squares covering the body (subdermal wounds). Each square on the skin was scored according to the affected area (score 0-4), intensity (score 0-4) and the degree of purulence (0-2), whereas each square on the body was scored for the affected area (score 0-3) and the intensity of injury (0-2). The bite score is then represented by the sum of all skin and body injuries and ranges from 0 to 300.

### Functional *In Vitro* GC Sensitivity Assay of Isolated Splenocytes

The isolation and *in vitro* stimulation of splenocytes for the GC sensitivity assay was done as previously described (14). Briefly, spleens were mechanically disrupted using a nylon cell strainer (70 µm; Corning, USA) and the plunger of a syringe to obtain a single cell suspension. Erythrocytes were removed by incubating the cell suspension for 2 min in lysis buffer (155 mM NH_4_Cl, 10 mM KHCO_3_, 10mM EDTA) followed by addition of Hanks’ Balanced Salt solution (HBSS; Sigma-Aldrich)/10% heat-inactivated fetal calf serum (FCS) to stop the lysis. Following one washing step with HBSS and filtration of the cell suspension through a 70 µM nylon cell strainer, cells were resuspended in ice-cold Roswell Park Memorial Institute Medium (RPMI-1640, Sigma-Aldrich, St. Louis, MO, USA) supplemented with 10% fetal bovine serum (FBS, Gibco^®^, Thermo Fisher Scientific, Waltham, MA, USA), 50 U/mL penicillin and 50 µg/mL streptomycin (Gibco^®^, Thermo Fisher Scientific) and counted using an automated cell counter (TC-20, Bio-Rad Laboratories, München, Germany). Cells were finally resuspended at a concentration of 5 x 10^6^ cells/ml and plated in flat-bottom, 96-well plates in a final volume of 100 µl/well (2.5 x 10^5^ cells/well). Cells either remained untreated or were stimulated with lipopolysaccharide (LPS, *Escherichia coli* O111:B4, final concentration 1µg/ml; Sigma-Aldrich, Deisenhofen, Germany). Moreover, various corticosterone (CORT; Sigma-Aldrich, Deisenhofen, Germany) concentrations (final concentration (days 8, 9, 15, 16 and 20 + 30d SHC): 0, 0.005, 0.05, 0.1, 0.5 and 5 µM respectively; final concentration (day 20): 0, 0.1 and 5 µM respectively), diluted in 95% ethanol, were added to the wells. Cells were incubated (37°C, 5% CO2) for 24 h (day 20) or 48 h (all other days) and cell viability was determined using a commercially available colorimetric assay (CellTiter 96 Aqueous One Solution Assay, Promega, Madison, WI). The included formazan is converted by living cells to a red dye, which can be measured by using an enzyme-linked immunosorbent assay (42) plate reader (Fluostar Optima, BMG Labtech, Offenburg, Germany) at 450 nm. The absorbance [optical density (OD)] of unstimulated cells was subtracted from the corresponding LPS-stimulated cells and the resulting delta OD was set to 100% for the 0 µM CORT condition. Of note, statistical post-hoc analysis only considered comparisons of the delta LPS-basal ODs at the 0.005, 0.05, 0.1, 0.5 and 5 µM CORT conditions with the delta LPS-basal ODs at the 0 µM condition. GC insensitivity is indicated by the inability of isolated and *in vitro* LPS stimulated splenocytes to respond to the immunosuppressive effects of CORT with a decrease in cell viability (19). Therefore, a significantly lower cell viability of the various CORT concentrations compared to the CORT = 0 µM condition (set to 100%) was considered as functional indication of glucocorticoid sensitivity. Additionally, delta cell viability in the presence of 0.1 µM CORT (with 0 µM CORT condition set to 100%) was used to compare GC sensitivity between the different time points of the CSC paradigm. Hereby, SHC mice of each time point were set to 100%, pooled and compared to CSC mice of each time point.

### Protein Extraction

Remaining splenocytes were used to isolate proteins for WB analysis. Protein extraction was performed according to the manufacturer’s instructions of a commercially-available protein extraction kit (Cell Extraction Buffer, Invitrogen, Vienna, Austria). Cells were washed with phosphate buffered saline (PBS) and then resuspended in the respective amount of cell extraction buffer (1 ml/10^8^ cells; Life Technologies GmbH, Darmstadt, Germany) supplemented with complete mini protease inhibitor (Roche Diagnostics GmbH, Mannheim, Germany). After 30 min of incubation on ice, cell suspensions were centrifuged and the supernatants were collected and stored at -20°C until analysis. The protein concentration was determined using a commercially-available quantification kit (Bicinchoninic Acid Protein Assay Kit, Thermo Scientific, Rockford, USA).

### Western Blot Analysis

Western blotting was performed as previously described (14). Briefly, equal amounts of protein lysates (15µg) from each experimental mouse were loaded on a 10% and 15% SDS gel (day 8, 9, 15, 16 and 20 + 30d SH) or on a 4-20% Mini-PROTEAN TGX Stain-Free Gel (Bio-Rad Laboratories, München, Germany; day 20), and blotted on nitrocellulose blotting membranes (GE Healthcare Life Sciences, Germany), respectively. All membranes were blocked with 5% milk in Tris-buffered saline containing Tween (TBS-T). Membranes derived from 10% SDS gels were cut at approximately 50 kDa according to the protein ladder (PageRuler Plus Prestained Protein Ladder, Thermo Scientific, Rockford, USA) and were incubated overnight (4°C) with primary antibodies for rabbit anti-mouse GR (1:1200, Santa Cruz Biotechnology, Inc., Heidelberg, Germany) and mouse anti-mouse glyceraldehyde 3-phosphate dehydrogenase (GAPDH; 1:5000, Thermo Scientific, Rockford, USA). After incubation with HRP-conjugated goat anti-rabbit secondary antibody (1:3000, Cell Signaling Technology, New England Biolabs GmbH, Frankfurt am Main, Germany) or horse anti-mouse secondary antibody (1:5000, Signaling Technology, New England Biolabs GmbH, Frankfurt am Main, Germany), membranes were developed using ClarityTM Western ECL Substrate (Bio-Rad Laboratories, München, Germany). Antibody binding was visualized on Molecular Imager^®^ ChemiDoc™ MP system (Bio-Rad Laboratories, München, Germany). Membranes derived from 15% SDS gels were cut at approximately 25 or 45 kDa according to the protein ladder and were incubated overnight (4°C) with primary antibodies for rabbit anti-mouse MIF (1:1200, abcam, Cambridge, United Kingdom) and rabbit anti-mouse CD11b (1:2000, abcam, Cambridge, United Kingdom). After incubation with HRP-conjugated goat anti-rabbit secondary antibody (MIF: 1:3000, CD11b: 1:2000, Cell Signaling Technology, New England Biolabs GmbH, Frankfurt am Main, Germany), the membranes were developed and antibody binding was visualized. The membranes were stripped using Re-Blot Plus Antibody Stripping Solution (Millipore GmbH, Schwalbach, Germany) and incubated with primary mouse anti-mouse glyceraldehyde 3-phosphate dehydrogenase (GAPDH; 1:5000, Thermo Scientific, Rockford, USA) for 1 h at room temperature. After incubation with HRP-linked horse anti-mouse secondary antibody (1:5000, Signaling Technology, New England Biolabs GmbH, Frankfurt am Main, Germany), the membranes were developed and the antibody binding was visualized. Membranes derived from Mini-PROTEAN TGX Stain-Free Gel were incubated overnight (4°C) with primary antibody for rabbit anti-mouse MIF (1:1200). After incubation with HRP-conjugated goat anti-rabbit secondary antibody (1:3000), the membranes were developed and antibody binding was visualized. Semiquantitative densitometric analyses of the signals were performed using Image Lab™ Software (Bio-Rad Laboratories, Munich, Germany). GR (~86 kDa), MIF (~ 11-13 kDa) and CD11b (~ 170 kDa) protein expression for each mouse was normalized to GAPDH expression (~ 36 kDa; days 8, 9, 15, 16 and 20 + 30d SH) or the amount of protein loaded onto the gel (day 20). Considering the latter, the amount of whole protein on Mini-PROTEAN TGX Stain-Free Gels was detected using a Molecular Imager^®^ ChemiDoc™ MP system (Bio-Rad Laboratories, München, Germany). Protein load of each sample was calculated as sum of representative protein bands in each lane as described earlier (43). No protein for Western Blot analysis was available from one SHC mouse euthanized on day 8 (SHC: n = 7 for relative GR, MIF and CD11b protein expression) and one SHC mouse euthanized on day 9 (SHC: n = 7 for relative GR, MIF and CD11b protein expression). Additionally, the relative GR protein expression of one CSC mouse euthanized on day 9 (CSC: n = 6 for relative GR protein expression) and two CSC mice euthanized on day 15 (CSC: n = 6 for relative GR protein expression) could not be calculated, as the GAPDH band was not detectable. Furthermore, no protein for relative GR protein expression was available from one SHC mouse euthanized on day 15 (SHC: n = 7 for relative GR protein expression).

### Statistics

For statistical analysis and graphical illustrations GraphPad Prism (version 8.4.2, GraphPad Software, LCC) was used. Kolmogorov-Smirnov test with Lilliefors’ correction was employed to test for normal distribution. Outliers in normally distributed data sets were identified by Grubbs test and excluded from further analysis (one outlier in the SHCs in absolute spleen weight on day 9, moreover, one outlier was detected in the correlational analysis between the splenic GC resistance at 0.005 µM CORT and the bite score as well as the GC resistance at 0.1 µM CORT, respectively). Normally distributed data sets were analyzed by parametric statistics, i.e. two-tailed Student’s *t*-tests or two-tailed Student’s *t*-tests with Welch’s correction when appropriate. Not normally distributed data sets were analyzed by non-parametric statistics, i.e. Mann-Whitney U test (MWU, one factor, two independent samples), Kruskal-Wallis test (KW, one factor, more than two independent samples) and Friedman analysis of variance (ANOVA) (one factor, more than two dependent samples). Correlational analyses were performed using Pearson correlation (both parameters normally distributed) or Spearman correlation (at least one parameter not normally distributed). All statistical tests comparing more than two samples were followed by post-hoc Dunn’s multiple comparison, when a significant main effect was found. Data are presented as bars (mean + SEM) with individual values. The level of significance was set at P ≤ 0.05.

## Results

### Bite Wounds

Statistical analysis revealed a significant main effect for the readout bite wounds (KW, H(5) = 17.66; P < 0.003; **Figure 1B**). In detail, while the bite score of CSC mice euthanized immediately after termination of CSC exposure on days 8, 9, 15, 16 and 20 was comparable, mice euthanized on day 9 (P = 0.017), 15 (P = 0.025), 16 (P = 0.009) and 20 (P = 0.045) showed significantly higher bite score compared with mice euthanized on day 51 (Day 20 of the CSC paradigm + 30 days of single housing).

### Effects of CSC on the Spleen at Day 8 of CSC

**Figure 2:**
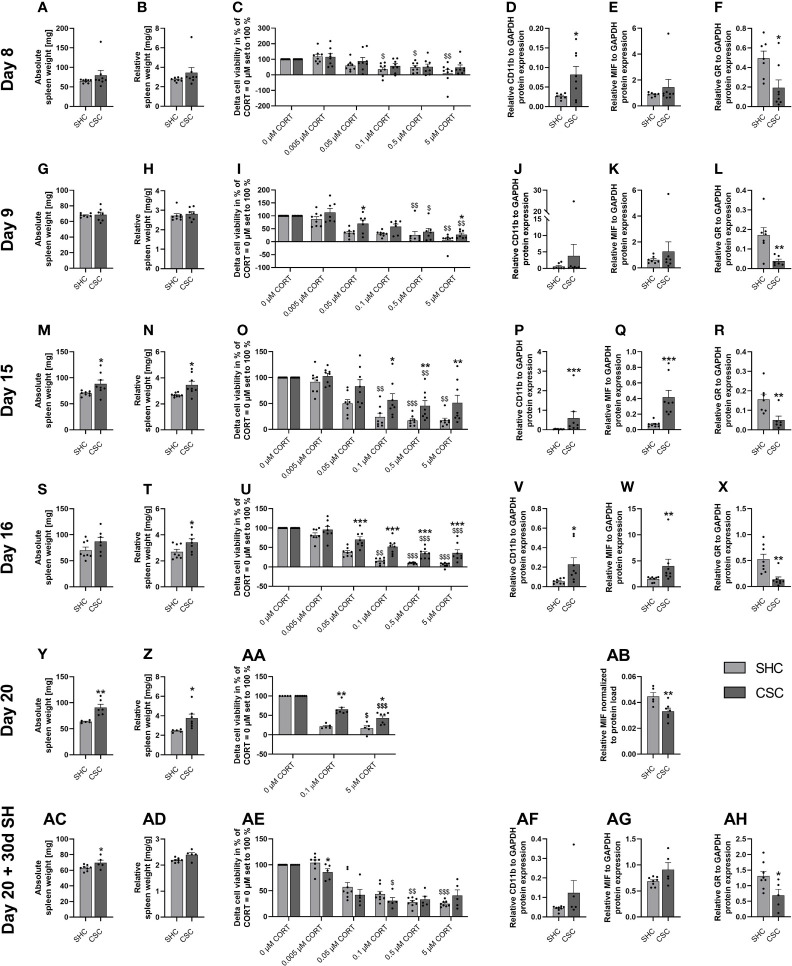
Days 8, 9, 15, 16, 20 of the chronic subordinate colony housing (CSC) paradigm or single housed control (SHC) condition and one month following termination of the CSC paradigm – effects on the spleen. **(A, G, M, S, Y, AC)** Absolute spleen weight. **(B, H, N, T, Z, AD)** Relative spleen weight. **(C, I, O, U, AA, AE)** Delta cell viability (Lipopolysaccharide (LPS)-stimulated minus basal conditions) of isolated and *in vitro* stimulated splenocytes with different concentrations of corticosterone (CORT; CORT = 0 µM set to 100%). **(D, J, P, V, AF)** Relative CD11b protein expression, **(E, K, Q, W, AB, AG)** relative macrophage migration inhibitory factor (MIF) protein expression and **(F, L, R, X, AH)** relative glucocorticoid receptor (GR) protein expression on day 8 **(A–F)**, day 9 **(G–L)**, day 15 **(M–R)**, day 16 **(S–X)** and day 20 **(Y–AB)** of the CSC paradigm and one month following termination of the CSC paradigm **(AC–AH)**. Data are presented as mean + SEM including individual values. * *P* ≤ 0.05, **P ≤ 0.01, ***P ≤ 0.001 versus respective SHC; ^$^ *P* ≤ 0.05, ^$$^ *P* ≤ 0.01, ^$$$^ *P* ≤ 0.001 versus respective 0 µM CORT condition. GAPDH, glyceraldehyde 3-phosphate dehydrogenase; SH, single housing.

Statistical analysis revealed that absolute (**Figure 2A**) and relative spleen weight (**Figure 2B**) were comparable between SHC and CSC mice on day 8 of CSC exposure. Moreover, a significant main effect of factor CORT was found for delta cell viability (LPS minus basal conditions) in isolated and *in vitro* stimulated splenocytes from both SHC (Friedman, X^2^ (5, 8) = 28.36; P < 0.001) and CSC (Friedman, X^2^ (5, 8) = 20.57; P < 0.001) mice (**Figure 2C**), with the 0.1 µM (P = 0.049), 0.5 µM (P = 0.032) and 5 µM CORT (P = 0.003) condition being significantly lower in SHC mice compared with the respective 0 µM CORT condition (set to 100%). Finally, isolated splenocytes from CSC mice showed an increased relative CD11b (*t*-test, P = 0.033; **Figure 2D**), unaffected relative MIF (**Figure 2E**) and decreased relative GR protein expression (MWU, P = 0.021; **Figure 2F**) compared with SHC.

### Effects of CSC on the Spleen at Day 9 of CSC

Statistical analysis revealed that absolute (**Figure 2G**) and relative spleen weight (**Figure 2H**) were comparable between SHC and CSC mice on day 9 of CSC exposure. Moreover, a significant main effect of factor CORT was found for delta cell viability (LPS minus basal conditions) in isolated and *in vitro* stimulated splenocytes from both SHC (Friedman, X^2^ (5, 8) = 27.14; P < 0.001) and CSC (Friedman, X^2^ (5, 7) = 25.04; P < 0.001) mice (**Figure 2I**), with the 0.5 µM (SHC: P = 0.005; CSC: P = 0.025) and 5 µM (SHC and CSC: P = 0.002) condition being significantly lower in both groups compared with the respective 0 µM CORT condition (set to 100%). Importantly, delta cell viability at the 0.05 µM (MWU, P = 0.054) and 5 µM CORT (MWU, P = 0.021) condition was significantly higher in CSC compared with respective SHC mice, indicating mild signs of GC resistance on day 9 of CSC. Finally, isolated splenocytes from CSC mice showed an unaffected relative CD11b (**Figure 2J**) and MIF (**Figure 2K**) expression, while relative GR protein expression (*t*-test, P = 0.011; **Figure 2L**) was decreased compared with SHC mice.

### Effects of CSC on the Spleen at Day 15 of CSC

Statistical analysis revealed that absolute (*t*-test, P = 0.040; **Figure 2M**) and relative spleen weight (*t*-test, P = 0.026; **Figure 2N**) were increased in CSC compared with SHC mice on day 15 of CSC exposure. Moreover, a significant main effect of factor CORT was found for delta cell viability (LPS minus basal conditions) in isolated and *in vitro* stimulated splenocytes from both SHC (Friedman, X^2^ (5, 8) = 35.00; P < 0.001) and CSC (Friedman, X^2^ (5, 8) = 25.14; P < 0.001) mice (**Figure 2O**), with the 0.5 µM (SHC: P < 0.001; CSC: P = 0.008) condition being significantly lower in both groups compared with the respective 0 µM CORT condition (set to 100%). In contrast, delta cell viability at the 0.1 µM (P = 0.008) and 5 µM (P = 0.002) condition was significantly lower compared with the respective 0 µM CORT condition (set to 100%) in SHC but not CSC mice. Together with a significantly increased delta cell viability at the 0.1 µM (MWU, P = 0.038), 0.5 µM (MWU, P = 0.007) and 5 µM CORT (MWU, P = 0.010) condition in CSC compared with respective SHC mice, this indicates moderated signs of GC resistance on day 15 of CSC. Finally, isolated splenocytes from CSC mice showed an increased relative CD11b (MWU, P < 0.001; **Figure 2P**) and MIF (MWU, P < 0.001; **Figure 2Q**) expression, while relative GR protein expression (MWU, P = 0.014; **Figure 2R**) was decreased compared with SHC mice.

### Effects of CSC on the Spleen at Day 16 of CSC

Statistical analysis revealed that absolute (**Figure 2S**) spleen weight was comparable between the groups, while relative spleen weight (*t*-test, P = 0.047; **Figure 2T**) was increased in CSC compared with SHC mice on day 16 of CSC exposure. Moreover, a significant main effect of factor CORT was found for delta cell viability (LPS minus basal conditions) in isolated and *in vitro* stimulated splenocytes from both SHC (Friedman, X^2^ (5, 8) = 37.14; P < 0.001) and CSC (Friedman, X^2^ (5, 8) = 32.29; P < 0.001) mice (**Figure 2U**), with the 0.5 µM (SHC and CSC: P < 0.001) and 5 µM (SHC and CSC: P < 0.001) condition being significantly lower in both groups compared with the respective 0 µM CORT condition (set to 100%). In contrast, delta cell viability at the 0.1 µM CORT condition was significantly lower compared with the respective 0 µM CORT condition (set to 100%) in SHC (P = 0.013) but not CSC mice. Together with a significantly increased delta cell viability at the 0.05 µM (MWU, P = 0.001), 0.1 µM (MWU, P < 0.001), 0.5 µM (MWU, P < 0.001) and 5 µM CORT (MWU, P < 0.001) condition in CSC compared with respective SHC mice, this indicates severe signs of GC resistance on day 16 of CSC. Finally, isolated splenocytes from CSC mice showed an increased relative CD11b (*t*-test, P = 0.039; **Figure 2V**) and MIF (MWU, P = 0.003; **Figure 2W**) expression, while relative GR protein expression (MWU, P = 0.002; **Figure 2X**) was decreased compared with SHC mice.

### Effects of CSC on the Spleen at Day 20

Statistical analysis revealed that absolute (*t*-test, P < 0.006; **Figure 2Y**) and relative (*t*-test, P < 0.019; **Figure 2Z**) spleen weight was significantly increased in CSC vs. SHC mice following 19 days of CSC. Moreover, a significant main effect of factor CORT was found for delta cell viability (LPS minus basal conditions) in isolated and *in vitro* stimulated splenocytes from both SHC (Friedman, X^2^ (2, 5) = 7.60; P = 0.024) and CSC (Friedman, X^2^ (2, 7) = 14.00; P < 0.001) mice (**Figure 2AA**), with the 5 µM (SHC: P = 0.034 and CSC: P < 0.001) condition being significantly lower in both groups compared with the respective 0 µM CORT condition (set to 100%). Additionally, delta cell viability at the 0.1 µM (MWU, P = 0.003) and 5 µM CORT (MWU, P = 0.018) condition was significantly increased in CSC compared with respective SHC mice. Furthermore, splenic MIF protein expression was found to be significantly decreased (*t*-test, P = 0.014; **Figure 2AB**) in CSC vs. SHC mice. Finally, absolute (SHC: 3.35 ± 0.09; CSC: 4.25 ± 0.11; *t*-test, P < 0.001) and relative (SHC: 0.129 ± 0.003; CSC: 0.159 ± 0.003; *t*-test, P < 0.001) adrenal weight were increased in CSC compared with SHC mice on day 20 of CSC.

### Effects of CSC on the Spleen at Day 51 (Day 20 of the CSC Paradigm + 30 Days of Single Housing)

Statistical analysis revealed that absolute (*t*-test, P = 0.053; **Figure 2AC**) but not relative spleen weight (**Figure 2AD**) was significantly increased in CSC vs. SHC mice 30 days following termination of the CSC paradigm on day 20. Moreover, a significant main effect of factor CORT was found for delta cell viability (LPS minus basal conditions) in isolated and *in vitro* stimulated splenocytes from both SHC (Friedman, X^2^ (5, 8) = 35.07; P < 0.001) and CSC (Friedman, X^2^ (5, 5) = 15.40; P = 0.009) mice (**Figure 2AE**), with the 0.5 µM (SHC: P = 0.013) and 5 µM (SHC: P < 0.001) CORT condition being significantly lower in the SHC group compared with the respective 0 µM CORT condition (set to 100%). In contrast, delta cell viability at the 0.1 µM condition was significantly lower compared with the respective 0 µM CORT condition (set to 100%) in CSC (P = 0.035) but not SHC mice. Together with a significantly lower delta cell viability at the 0.005 µM (MWU, P = 0.030) condition in CSC compared with respective SHC mice, this indicates a slightly increased GC sensitivity on day 30 following termination of CSC. Finally, isolated splenocytes from CSC mice showed a comparable relative CD11b (**Figure 2AF**) and relative MIF (**Figure 2AG**) and decreased relative GR (*t*-test, P = 0.028; **Figure 2AH**) protein expression compared with respective SHC mice. Additionally, absolute (SHC: 3.11 ± 0.09; CSC: 3.60 ± 0.07; *t*-test, P = 0.004) and relative (SHC: 0.109 ± 0.004; CSC: 0.126 ± 0.004; MWU, P = 0.011) adrenal weight was increased in CSC compared with SHC mice 30 days following CSC termination.

### Correlational Analyses

**Figure 3:**
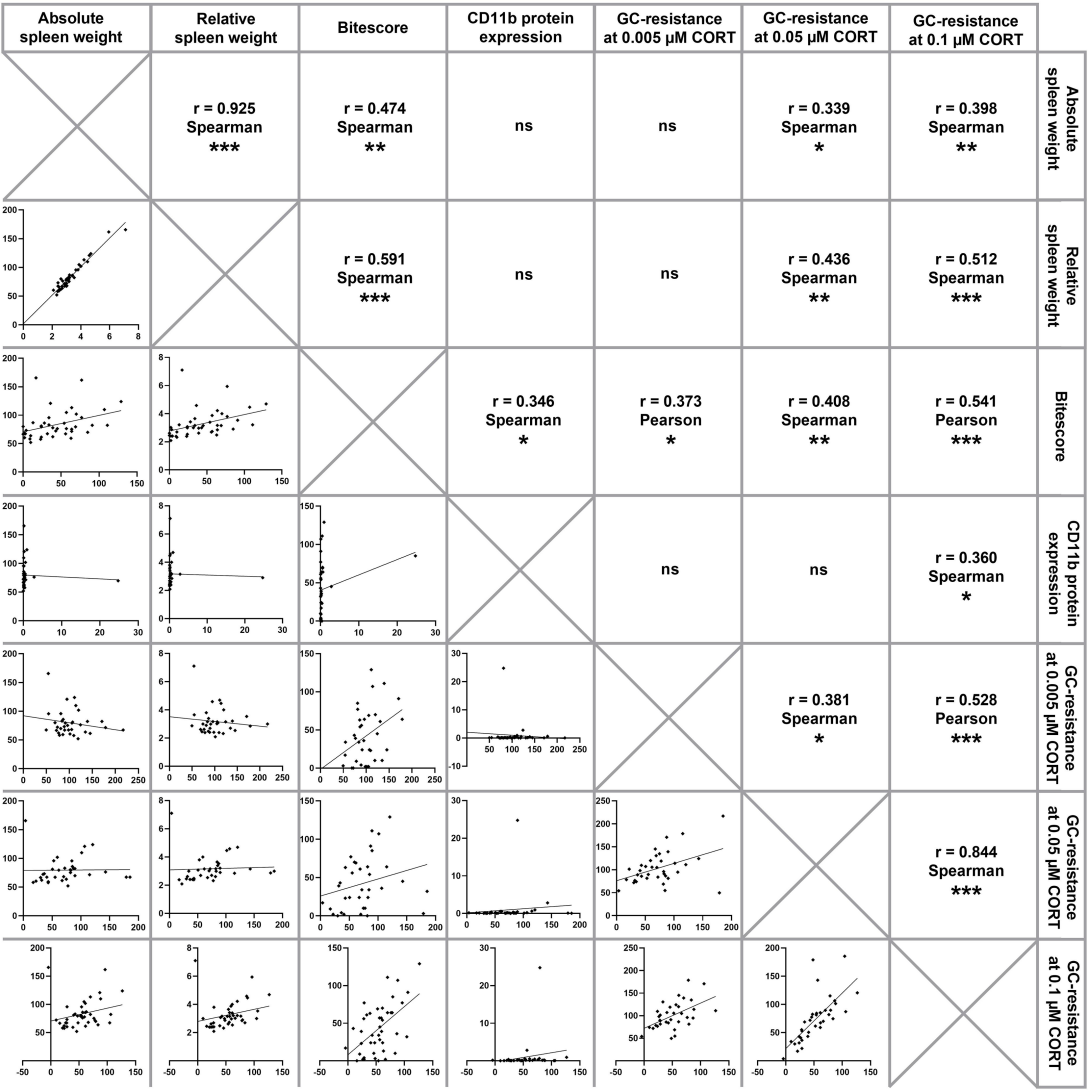
Correlation analysis. Depicted are correlations between absolute and relative spleen weight, bite score, splenic CD11b protein expression and functional splenic glucocorticoid (GC) resistance at 0.005 µM, 0.05 µM and 0.1 µM corticosterone (CORT), respectively. Of note, CSC mice of all time points have been pooled for running correlational analyses, which were done using either Pearson correlations (both parameters normally distributed) or Spearman correlations (at least one parameter not normally distributed). Significant correlations are indicated by *P ≤ 0.05, **P ≤ 0.01, ***P ≤ 0.001, ns, not significant.

Statistical analyses considering all CSC mice of the present study (**Figure 3**) revealed a positive correlation between the absolute and relative spleen weight (Spearman, rho = 0.925; P < 0.001). Moreover, both, absolute and relative spleen weight correlated positively with splenic GC insensitivity at 0.05 µM CORT (absolute spleen weight (Spearman, rho = 0.339; P = 0.046); relative spleen weight (Spearman, rho = 0.436; P = 0.009)) and 0.1 µM CORT (absolute spleen weight (Spearman, rho = 0.398; P = 0.009); relative spleen weight (Spearman, rho = 0.512; P = 0.001)) as well as the bite score (absolute spleen weight (Spearman, rho = 0.474; P = 0.002); relative spleen weight (Spearman, rho = 0.591; P < 0.001)), respectively. The bite score correlated positively with splenic CD11b protein expression (Spearman, rho = 0.346; P = 0.039) and splenic GC insensitivity at 0.005 µM CORT (Pearson, rho = 0.373; P = 0.027), 0.05 µM CORT (Spearman, rho = 0.408; P = 0.014) and 0.1 µM CORT (Pearson, rho = 0.541; P < 0.001). A positive correlation was also found between splenic GC insensitivity at 0.1 µM CORT and splenic CD11b protein expression (Spearman, rho = 0.360; P = 0.031). Furthermore, a positive correlation was found between splenic GC insensitivity at 0.1 µM CORT and GC insensitivity at 0.005 µM CORT (Pearson, rho = 0.528; P = 0.001) as well as GC insensitivity at 0.05 µM CORT (Spearman, rho = 0.844; P < 0.001), respectively. In addition, GC insensitivity at 0.005 µM CORT correlated positively with GC insensitivity at 0.05 µM CORT (Spearman, rho = 0.381; P = 0.022).

### Analysis of CSC-Induced GC Resistance Throughout All Time Points Assessed

**Figure 4:**
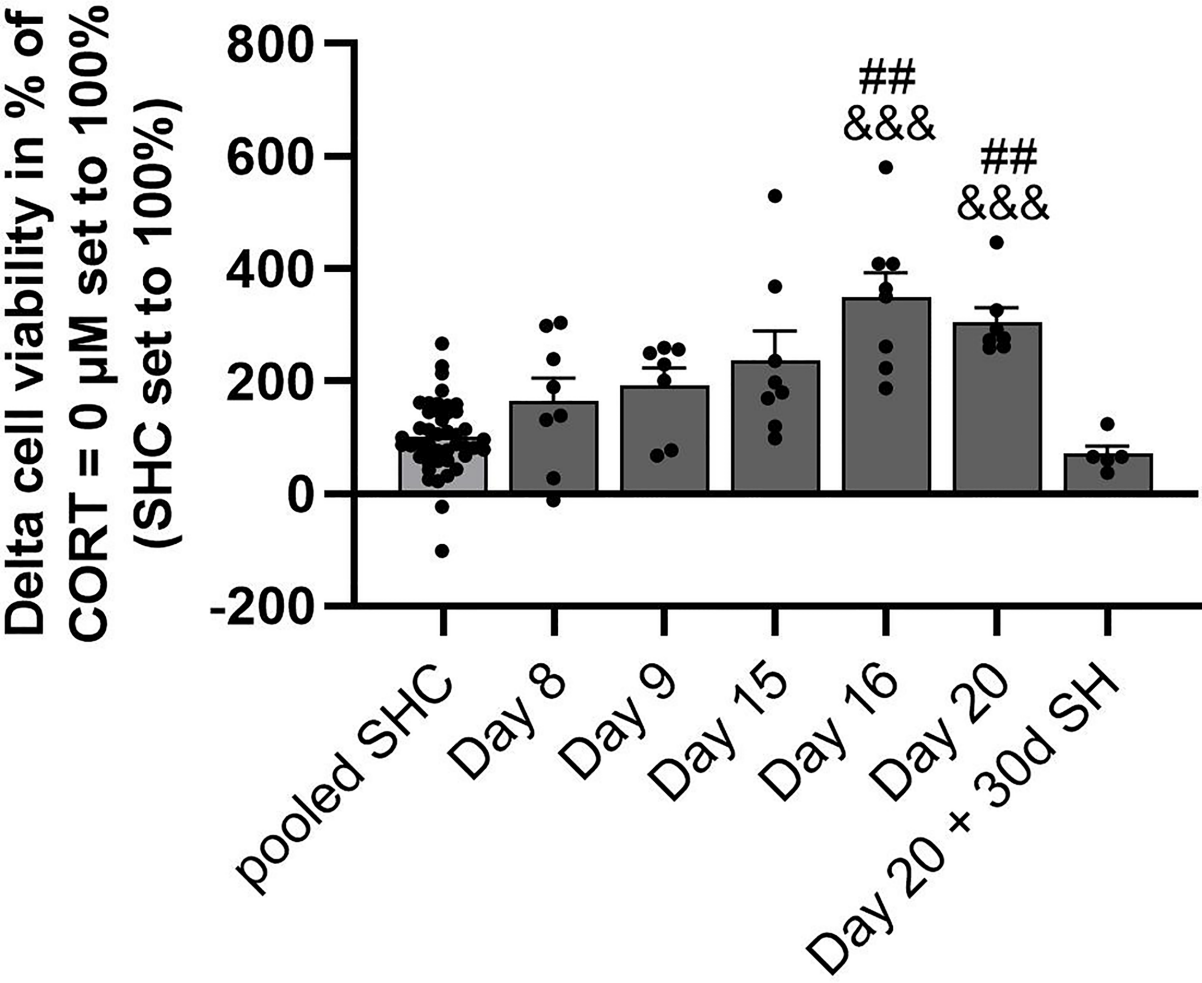
Analysis of CSC-induced GC resistance throughout all time points assessed. Depicted is the delta cell viability (Lipopolysaccharide (LPS)-stimulated minus basal conditions) of isolated and *in vitro* stimulated splenocytes of CSC and SHC mice at 0.1 µM corticosterone (CORT; CORT = 0 µM set to 100%) concentration. The single-housed control (SHC) mice of each time point were set to 100%. Data are presented as mean + SEM including individual values. ^##^ *P* ≤ 0.01 *versus* day 20 + 30d of single housing (SH); ^&&&^ *P* ≤ 0.001 *versus* pooled SHC.

Statistical analysis revealed a significant main effect of *in vitro* LPS-induced GC resistance in the presence of 0.1 µM CORT (with CORT = 0 µM set to 100%) over all time points (SHCs set to 100%; KW, H(6) = 43.72; P < 0.001; **Figure 4**). In detail, CSC mice euthanized on days 16 and 20 showed a significantly higher delta cell viability when compared to SHC mice pooled from all time points (Day 16: P < 0.001; Day 20: P < 0.001) and mice euthanized one month after termination of the CSC paradigm (Day 16: P = 0.002; Day 20: P = 0.003), respectively.

## Discussion

In the present study we confirm own earlier findings showing that mice exposed to the CSC paradigm develop splenomegaly, increased splenic CD11b and decreased GR protein expression, as well as severe functional GC insensitivity during *in vitro* stimulation of isolated spleen cells with LPS (14, 15, 19, 22). In extension of these findings we show that these are transient stress consequences, developing gradually over time during chronic psychosocial stressor exposure and normalizing almost completely within 30 days following termination of stressor exposure. Moreover, our data are in line with and extend previous findings (14) by showing that a fast and long-lasting decrease in splenic GR expression is not sufficient to induce functional splenic *in vitro* GC insensitivity. Although an increase in splenic MIF expression paralleling the development of severe functional splenic *in vitro* GC insensitivity following two weeks of CSC exposure further suggests that MIF at least in part might be involved in mediating CSC-induced functional spleen changes, further studies are required as splenic MIF expression was decreased in CSC compared with SHC mice on day 20 of CSC despite pronounced splenomegaly and functional GC resistance.

The best biomarker for classification and class prediction of stress in the CSC mouse model is an increased adrenal weight (44). In line with this, CSC mice used in the current study had significantly enlarged adrenals compared to respective SHC mice, as reported just recently by our group for days 8, 9, 15 and 16 of CSC exposure (38) or in the current study for day 20 and day 20 + 30d SH, clearly indicating that all CSC mice included in the current study were chronically stressed. In accordance with earlier studies done by us and others employing a chronic psychosocial stressor allowing direct physical contact, exposure to the CSC paradigm resulted in an increased spleen weight and functional splenic GC insensitivity when stimulated with LPS *in vitro* (14–16, 18, 19, 21, 22, 24). The current study extends these findings by providing a time course of relevant CSC-induced spleen changes. While splenomegaly was absent on days 8 and 9 of CSC, spleens of CSC mice were significantly enlarged compared with respective SHC mice on days 15, 16 and 20 of chronic psychosocial stressor exposure. In accordance, the severity of functional splenic GC insensitivity in response to *in vitro* LPS stimulation was only mild on days 8 and 9, but strongly increased on days 15, 16 and 20 of CSC, respectively. In line with the latter, CSC mice euthanized on days 16 and 20 showed a significantly higher delta cell viability in the presence of 0.1 µM CORT compared to SHC mice pooled from all time points and CSC mice euthanized one month after termination of the CSC paradigm, indicating a pronouced functional *in vitro* GC resistance on day 16 and 20 of the CSC paradigm. A link between stress-induced splenomegaly and the development of functional splenic *in vitro* GC insensitivity is supported by the positive correlation of relative and absolute spleen weight with the severity of GC resistance found in the present and also in earlier studies (14, 15). Furthermore, besides a positive correlation of the absolute with the relative spleen weight, we found that GC resistance at different concentrations of CORT showed a strong positive correlation with each other. A strong positive correlation of both spleen weight and functional splenic GC insensitivity with the severity of bite wounds received during a 19-day CSC exposure is in line with our previous findings and further suggests a critical role of wounding in social stress-induced spleen consequences (14). This hypothesis is also supported by our recent study showing that abdominal surgery prior to chronic psychosocial stress in a bite wound-independent manner promotes splenomegaly and functional *in vitro* GC resistance (19). Furthermore, while mice exposed to the social reorganization, the paired fighting and the social disruption stress paradigm, which all hold a social component allowing direct physical interaction and, thus, wounding, develop a myeloid cell-induced splenomegaly and GC insensitivity of isolated and *in vitro* LPS-stimulated splenocytes, these effects were absent in mice exposed to restraint stress lacking a social component (18, 21, 23, 24). In extension of this concept, data of the present study suggest that continuous psychosocial stress induced by the CSC model in combination with wounding requires at least 1-2 weeks until translating into a severe splenomegaly and functional splenic *in vitro* GC resistance. The latter is indicated by the fact that severe splenomegaly and functional splenic *in vitro* GC resistance were detectable only on days 15, 16 and 20 of CSC exposure, despite the bite score was comparable between all CSC groups on days 8, 9, 15, 16 and 20. Of note, to put the term “severe” into perspective, adrenalectomized C57BL6/J mice exhibit splenomegaly with spleens weighing 130-150 mg/mouse (45). However, although it is likely that wounding occurs within the first hours of hierarchy establishment after exposing CSC mice to the respective dominant aggressor mice on days 1, 8 and 15, a bite score quantifying overall wounding severity only in euthanized mice (14, 19) does not allow to delineate when exactly wounding has happened. Therefore, an alternative bite score assessing the severity of wounding in a daily manner in alive mice during CSC exposure is required to exclude that CSC mice euthanized on days 15, 16 or 20 of CSC showing severe splenomegaly and functional splenic *in vitro* GC resistance were wounded just shortly before euthanasia. However, as both splenomegaly and functional splenic *in vitro* GC resistance were only slightly pronounced 24 h after exposure to the second aggressor mouse (i.e. day 9) but severely pronounced after 7 days of cohousing with the second aggressor mouse, CSC-induced spleen changes seem to develop gradually over time of stressor exposure and to be independent of the dominant aggressor changes on days 8 and 15. Importantly, the time course of the here described spleen effects matches nicely with the time course reported recently for development of adrenal *in vitro* insensitivity to ACTH (38), suggesting that chronic stress-induced adaptations at the HPA axis and spleen level develop concomitantly following two weeks of continuous stressor exposure. Support for the idea that the above described spleen changes as a consequence of chronic stress and severe wounding represent a positive adaptation rather than a maladaptive functional breakdown comes from studies showing that CD11b^+^ macrophages produce important chemo-attractants and growth factors (46), ensuring proper wound healing during phases of chronically elevated levels of immunosuppressive CORT, as for instance chronic psychosocial stress exposure. In line with this interpretation, CSC-induced splenomegaly and functional splenic GC insensitivity in response to *in vitro* LPS stimulation is only a transient phenomenon, normalizing almost completely during 30 days of recovery from CSC and wound healing as suggested by a significantly lower bite score compared with days 9, 15, 16 and 20 during CSC. Given the fact that absolute spleen weight of CSC mice euthanized 30 days following termination of CSC was still slightly increased, we can exclude that this set of CSC mice were not sufficiently wounded during CSC and consequently did not at all develop typical CSC-induced changes in the spleen. However, as described above, an alternative bite score assessing the severity of wounding in a daily manner in alive mice during CSC exposure would be helpful to elucidate the exact timeline of wound healing following termination of the CSC paradigm. Support for the hypothesis that typical spleen effects seen in wounded CSC mice are transient comes from studies employing the SDR paradigm, showing that stress-induced GC resistance is still present 10 but not 30 days following 6 cycles of SDR exposure (21).

In line with previous data showing that the percentage of CD11b^+^ splenocytes is increased in CSC mice and that depletion of these CD11b^+^ splenocytes prevents CSC-induced functional GC resistance in isolated and *in vitro* LPS-stimulated splenocytes (14), statistical analysis considering all CSC mice of the present study revealed positive correlations of the bite score and GC resistance with splenic CD11b protein expression. The latter is consistent with findings reported in mice exposed to the SDR paradigm (18, 29) and the previously summarized hypothesis that psychosocial stressor exposure in combination with physical injury results in invasion of primed bone marrow derived and GC resistant CD11b^+^ cells into the spleen, resulting in splenomegaly and functional splenic *in vitro* GC resistance in the presence of LPS (15). In detail, while splenic CD11b protein expression was only slightly increased or not affected on days 8 and 9 of CSC and day 20 + 30d SH when CSC-induced splenomegaly and splenic GC resistance were absent or only of mild severity, splenic CD11b protein expression was strongly increased on days 15 and 16, when CSC-induced splenomegaly and splenic GC resistance were most pronounced. One limitation of the current study is that the CD11b Western Blot data does not allow any conclusions to be drawn on whether the CSC-induced increase in splenic CD11b expression is mediated by an increased CD11b expression per cell or by an increased number of CD11b^+^ splenocytes. However, in support of the latter we recently showed using flow cytometry that on day 20 of CSC exposure CSC vs. SHC mice are characterized by a pronounced decrease in the number of splenic B and T cells, paralleled by an increase in CD11b^+^ myeloid cells (14).

Although data collected in this and own previous studies clearly support a critical role for CD11b^+^ cells in CSC-induced GC resistance of isolated and *in vitro* LPS stimulated splenocytes, the underlying molecular mechanisms are not well understood. The only conclusion that can be drawn at the moment is that the decreased splenic GR protein expression found in CSC mice of the current and own previous studies (14) seems not to be involved in mediating these effects. This is indicated by the fact that splenic GR protein expression was downregulated in CSC versus SHC mice at days 8, 9, 15, 16 and 20 + 30d SH in the current study, as well as on day 20 assessed in a previous study (14), whereas severe splenomegaly and functional GC resistance of *in vitro* LPS stimulated splenocytes was only detectable on days 15, 16 and 20 of CSC. Given that splenic CD11b protein expression (except on day 8) was also not increased in the early phase of CSC and after termination of CSC, suggesting a lack of spleen invading GC resistant CD11b^+^ cells on these days, we hypothesize that splenic GR downregulation affects rather residual spleen cells than invading CD11b^+^ cells and, thus, does not play a major role in CSC-induced GC resistance of isolated CD11b^+^ splenocytes in the presence of LPS. Support for the idea that the decreased splenic GR expression, even if it not only affects residual spleen cells but also invading CD11b^+^ cells, is at least not sufficient to mediate CSC-induced GC resistance, comes from the fact that isolated splenocytes from CSC mice are GC sensitive during *in vitro* culturing in the absence of LPS (14). Of importance in this context are findings from Quan and colleagues, showing that functional splenic GC resistance in mice exposed to the SDR paradigm is paralleled by a compromised upregulation of nuclear GR protein expression and its subsequent nuclear translocation in response to *in vitro* LPS stimulation in CD11b^+^ splenocytes, resulting in a lack of transcriptional suppression of LPS-induced nuclear factor ‘kappa-light-chain-enhancer’ of activated B-cells (NF-κB) (16). As splenic GR protein expression is also reduced in SDR mice not exposed to LPS *in vitro* (16), similar to what we report for CSC mice, a compromised GR upregulation and GR nuclear translocation in response to LPS is likely to also play a role in CSC-induced GC resistance. However, further studies are needed to address this in detail.

Importantly, one factor known to drive NF-κB activation *via* a positive feedback loop (47) and to promote GC resistance indirectly *via* inhibiting GC-induced leucine zipper and subsequent MAP kinase phosphatase (MKP)-1 (GILZ) induction (48) is the pro-inflammatory cytokine MIF. A role of MIF in CSC-induced functional GC resistance in isolated and LPS-stimulated splenocytes in the present study is suggested by the fact that splenic MIF protein expression was increased in CSC versus SHC mice on days 15 and 16 of CSC, when severe splenomegaly and functional splenic GC insensitivity were detectable the first time. Although detailed mechanistic follow-up studies are required, we assume that increased splenic MIF levels at days 15 and 16 are mediated by an accumulating number of spleen invading macrophages. The latter are able to produce significant amounts of MIF (49) and play an important role in mediating SDR-induced GC resistance (21). As MIF moreover inhibits macrophage migration (50, 51), but in turn promotes neutrophil accumulation in inflamed tissues (52), we hypothesize that the decreased splenic MIF expression in CSC versus SHC mice on day 20, despite severe splenomegaly and functional splenic GC insensitivity, is indicating a switch from CSC-induced early invasion of GC-resistant macrophages to late invasion of GC-resistant neutrophils. Nevertheless, given that elevated levels of splenic MIF in the second week of CSC are accompanied by the development of adrenal insensitivity to ACTH (38), and that GCs rather induce than inhibit MIF production in macrophages (32), we cannot exclude CSC-induced adrenal changes to be involved in the above described findings.

Together with previous data, the findings of the current study support the hypothesis that psychosocial stressor exposure in mice promotes CD11b^+^ cell-mediated splenomegaly, at least when social stress allows direct physical contact and goes along with sufficient wounding. Moreover, our data suggest that this is progressively developing over at least one or two weeks of continuous stressor exposure, and represent a transient rather than a permanent phenomenon. Furthermore, CSC-induced reduction in splenic GR expression seems to be mediated by residual spleen cells rather than by invading CD11b^+^ cells and is not sufficient to mediate CSC-induced functional splenic GC resistance. Finally, our findings suggest MIF to play a role in CSC-induced splenic functional GC resistance, although further mechanistic studies have to address this in detail.

## Data Availability Statement

The raw data supporting the conclusions of this article will be made available by the authors, without undue reservation.

## Ethics Statement

The animal study was reviewed and approved by Regierungspraesidium Tuebingen, Germany.

## Author Contributions

SR, AW, and SK planned the study. SK, AW, HD, and EK performed the experiments. EK did the statistical analysis. SR, EK, MA, and DL wrote the manuscript. All authors contributed to the article and approved the submitted version.

## Funding

Parts of the presented work in this article were supported by the Collaborative Research Centre CRC1149 funded by the Deutsche Forschungsgemeinschaſt (DFG, German Research Foundation) - Project ID 251293561.

## Conflict of Interest

The authors declare that the research was conducted in the absence of any commercial or financial relationships that could be construed as a potential conflict of interest.

## Publisher’s Note

All claims expressed in this article are solely those of the authors and do not necessarily represent those of their affiliated organizations, or those of the publisher, the editors and the reviewers. Any product that may be evaluated in this article, or claim that may be made by its manufacturer, is not guaranteed or endorsed by the publisher.
